# Clinical Characteristics and Outcomes of Patients With *Cutibacterium acnes* Endocarditis

**DOI:** 10.1001/jamanetworkopen.2023.23112

**Published:** 2023-07-12

**Authors:** Floris J. Heinen, Florent Arregle, Floris S. van den Brink, Nina Ajmone Marsan, Lucas Bernts, Patrick Houthuizen, Otto Kamp, Nienke Roescher, Naomi Timmermans, Nelianne Verkaik, Jolien Roos-Hesselink, Marco C Post, Gilbert Habib, Wilco Tanis

**Affiliations:** 1 Department of Cardiology, Haga Hospital, Den Haag, the Netherlands; 2 Department of Cardiology, Assistance Publique–Hôpitaux de Marseille, La Timone Hospital, Marseille, France; 3 Department of Cardiology, Leiden University Medical Center, Leiden, the Netherlands; 4 Department of Medical Microbiology, Amsterdam UMC, Amsterdam, the Netherlands; 5 Department of Cardiology, Catharina Hospital, Eindhoven, the Netherlands; 6 Department of Cardiology, Amsterdam UMC, Amsterdam, the Netherlands; 7 Department of Medical Microbiology, St Antonius Hospital, Nieuwegein, the Netherlands; 8 Department of Cardiothoracic Surgery, Catharina Hospital, Eindhoven, the Netherlands; 9 Department of Medical Microbiology, Erasmus University Medical Center, Rotterdam, the Netherlands; 10 Department of Cardiology, Erasmus University Medical Center, Rotterdam, the Netherlands; 11 Department of Cardiology, Utrecht University Medical Center, Utrecht, the Netherlands; 12 Department of Cardiology, St Antonius Hospital, Nieuwegein, the Netherlands

## Abstract

**Importance:**

It is suggested that patients with *Cutibacterium acnes* endocarditis often present without fever or abnormal inflammatory markers. However, no study has yet confirmed this statement.

**Objective:**

To assess the clinical characteristics and outcomes of patients with *C acnes* endocarditis.

**Design, Setting, and Participants:**

A case series of 105 patients presenting to 7 hospitals in the Netherlands and France (4 university hospitals and 3 teaching hospitals) with definite endocarditis according to the modified Duke criteria between January 1, 2010, and December 31, 2020, was performed. Clinical characteristics and outcomes were retrieved from medical records. Cases were identified by blood or valve and prosthesis cultures positive for *C acnes*, retrieved from the medical microbiology databases. Infected pacemaker or internal cardioverter defibrillator lead cases were excluded. Statistical analysis was performed in November 2022.

**Main Outcomes and Measures:**

Main outcomes included symptoms at presentation, presence of prosthetic valve endocarditis, laboratory test results at presentation, time to positive results of blood cultures, 30-day and 1-year mortality rates, type of treatment (conservative or surgical), and endocarditis relapse rates.

**Results:**

A total of 105 patients (mean [SD] age, 61.1 [13.9] years; 96 men [91.4%]; 93 patients [88.6%] with prosthetic valve endocarditis) were identified and included. Seventy patients (66.7%) did not experience fever prior to hospital admission, nor was it present at hospitalization. The median C-reactive protein level was 3.6 mg/dL (IQR, 1.2-7.5 mg/dL), and the median leukocyte count was 10.0 × 10^3^/µL (IQR, 8.2-12.2 × 10^3^/µL). The median time to positive blood culture results was 7 days (IQR, 6-9 days). Surgery or reoperation was indicated for 88 patients and performed for 80 patients. Not performing the indicated surgical procedure was associated with high mortality rates. Seventeen patients were treated conservatively, in accordance with the European Society of Cardiology guideline; these patients showed relatively high rates of endocarditis recurrence (5 of 17 [29.4%]).

**Conclusions and Relevance:**

This case series suggests that *C acnes* endocarditis was seen predominantly among male patients with prosthetic heart valves. Diagnosing *C acnes* endocarditis is difficult due to its atypical presentation, with frequent absence of fever and inflammatory markers. The prolonged time to positivity of blood culture results further delays the diagnostic process. Not performing a surgical procedure when indicated seems to be associated with higher mortality rates. For prosthetic valve endocarditis with small vegetations, there should be a low threshold for surgery because this group seems prone to endocarditis recurrence.

## Key Points

**Question:**

What are the clinical characteristics and outcomes of patients with *Cutibacterium acnes* endocarditis?

**Findings:**

In this case series including 105 patients, *C acnes* endocarditis was seen predominantly among male patients with prosthetic heart valves and was characterized by absence of fever, close to normal inflammatory markers, and a prolonged time to positive blood culture results. Surgical treatment was required in a high proportion of patients.

**Meaning:**

This study suggests that *C acnes* endocarditis is difficult to diagnose because typical endocarditis features are often absent; it is recommended to extend blood culturing for male patients with new-onset prosthetic valve dysfunction, peripheral embolization, or signs of heart failure.

## Teaser

This case series assesses the clinical characteristics and outcomes of patients with *Cutibacterium acnes* endocarditis.

## Introduction

*Cutibacterium acnes* is a gram-positive bacterium, most known for its role in acne vulgaris. *C acnes* is generally considered nonpathogenic, and positive blood culture results are often regarded as contaminated.^1^ In recent years, more attention has been paid to *C acnes* as a cause of infective endocarditis (IE). Experienced clinicians hypothesize that patients with *C acnes* endocarditis are difficult to diagnose because typical IE features, such as fever and high levels of inflammatory markers, are often absent. However, these statements are supported only by case reports or small case series.^2,3^ Therefore, the aim of this study is to examine the clinical characteristics and outcomes of patients with *C acnes* IE in a large cohort of more than 100 patients.

## Methods

We performed a retrospective case series in 7 hospitals in the Netherlands and France (4 university hospitals and 3 teaching hospitals). Patients with a diagnosis of definite IE according to the modified Duke criteria^4^ between January 1, 2010, and December 31, 2020, were included. Cases with an isolated infected pacemaker or internal cardioverter defibrillator lead cases were excluded because we were interested only in valve endocarditis. Cases were identified by positive blood or valve and prosthesis culture results, retrieved from the medical microbiology databases. The Medical Ethics Review Committee Leiden The Hague Delft waived the need for consent because of the retrospective nature of this study in which participants are not subjected to any form of action. In addition, data were deidentified. Recurrence of IE was defined as IE relapse and/or a new episode of *C acnes* IE after initial treatment. Data are presented following the reporting guideline for case series.^5^

### Statistical Analysis

Statistical analysis was performed in November 2022 using IBM SPSS Statistics, version 27 (IBM Corp). Normally distributed variables were reported as mean (SD) values, and nonnormally distributed variables are displayed as median (IQR) values. Frequencies and percentages were calculated. There were no statistical tests performed.

## Results

A total of 105 patients (mean [SD] age, 61.1 [13.9] years; 96 men [91.4%]) with *C acnes* IE were identified and included. Most patients (94 [89.5%]) had previous cardiac surgery, which in most cases concerned previous valve surgery (93 [88.6%]). Baseline characteristics, including the types of previous cardiac surgical procedures, are presented in Table 1. The median time between index surgery and hospital presentation was 35.0 months (IQR, 15.5-71.5 months). There were 12 cases (11.4%) of native valve endocarditis: 5 aortic valve, 3 mitral valve, 1 combined aortic valve and mitral valve, and 3 tricuspid valve cases. The 3 patients with tricuspid valve native valve endocarditis had pacemaker leads. Tricuspid valve plasty was required for 1 patient with severe tricuspid valve regurgitation. For other native valve endocarditis cases, there was no underlying predisposition.

**Table 1.** Baseline Characteristics of the Total Cohort

Characteristic	Total cohort, No. (%) (N = 105)
Sex	
Male	96 (91.4)
Female	9 (8.6)
Age, mean (SD), y	61.1 (13.9)
Atrial fibrillation	40 (38.1)
Previous cerebrovascular disease	35 (33.3)^a^
Previous stroke	17 (16.2)
Previous transient ischemic attack	21 (20.0)
Left ventricular ejection fraction	
Good	83 (79.0)
Moderate	19 (18.1)
Poor	3 (2.9)
Immunocompromised	0
Previous *Cutibacterium acnes* endocarditis	2 (1.9)
Previous cardiac surgery	94 (89.5)
Aortic valve replacement	66 (62.9)
Bentall procedure	17 (16.2)
Mitral valve replacement	7 (6.7)
Mitral and tricuspid valve repair with annuloplasty ring	1 (1.0)
Isolated ascending aorta replacement	2 (1.9)
Isolated coronary artery bypass grafting	1 (1.0)

^a^ Five patients had a history of both stroke and transient ischemic attack.

At presentation, 70 patients (66.7%) did not report having experienced fever (defined as a measured temperature >38.0 °C) prior to hospital admission, nor was it present on physical examination (Table 2). The most frequently observed symptoms were dyspnea (38 [36.2%]), malaise (26 [24.8%]), and stroke (20 [19.0%]). Most patients (43 [41.0%]) experienced symptoms for 1 to 2 weeks prior to hospital admission. Five patients (4.8%) with prosthetic valve endocarditis (PVE) were asymptomatic, but routine echocardiography revealed new-onset prosthetic valve dysfunction. The median C-reactive protein (CRP) level at presentation was 3.6 mg/dL (IQR, 1.2-7.5 mg/dL (reference range, <0.1 mg/dL [to convert to milligrams per liter, multiply by 10.0]).^6^ Moreover, the CRP level was not suspected for a bacterial infection (≤1.0 mg/dL) for 23 patients (21.9%). The median leukocyte count was within the reference range (10.0 × 10^3^/µL [IQR 8.2-12.2 × 10^3^/µL] [to convert to ×10^9^/L, multiply by 0.001]). Laboratory test results at hospital admission are presented in Table 2. Blood cultures were performed for 87 patients, of whom 76 patients had at least 1 positive blood culture result. The median time to positive blood cultures was 7 days (IQR, 6-9 days), and 25.0% (19 of 76) became positive within 5 days. In total, 477 sets of blood cultures were obtained, of which 191 (anaerobic) cultures (40.0%) became positive.

**Table 2.** Admission Parameters of the Total Cohort

Parameter	Total cohort, No. (%) (N = 105)
Onset of symptoms	
≤2 wk	43 (41.0)
2-4 wk	18 (17.1)
5 wk to 3 mo	18 (17.1)
>3 mo	26 (24.8)
Symptoms	
Fever	35 (33.3)
Stroke	20 (19.0)
Myocardial infarction	3 (2.9)
Major arterial emboli	3 (2.9)
Malaise	26 (24.8)
Chills	38 (36.2)
Dyspnea	38 (36.2)
Weight loss	14 (13.3)
Newly diagnosed cardiac murmur	13 (12.4)
Splinter hemorrhage	2 (1.9)
Asymptomatic	8 (7.6)
Hemoglobin level, median (IQR), g/dL	7.6 (6.8-8.3)
MCV, median (IQR), µm^3^	86.0 (83.0-90.0)
CRP, median (IQR), mg/dL	3.6 (1.2-7.5)
CRP ≤1.0 mg/dL	23 (21.9)
Leukocyte count, median (IQR), ×10^3^/µL	10.0 (8.2-12.2)
eGFR, median (IQR), mL/min/1.73^2^	64.0 (59.0-87.0)

Abbreviations: CRP, C-reactive protein; eGFR, estimated glomerular filtration rate; MCV, mean corpuscular volume.

SI conversion factors: To convert hemoglobin to grams per liter, multiply by 10.0; MCV to femtoliters, multiply by 1.0; CRP to milligrams per liter, multiply by 10.0; and leukocytes to ×10^9^/L, multiply by 0.001.

In total, 64 patients were classified as having definite IE according to the modified Duke criteria.^4^ A positive blood culture result was a major criterion for 54 patients, and echocardiography findings were positive for all of these patients. The minor criterion observed most frequently was cardiac predisposition (63 of 64 [98.4%]). Peripheral embolization was observed for 30 of 64 patients (46.9%). Cardiac computed tomography (CT) was performed for 28 patients, with positive results for 26 patients (92.9%). ^18^Fluorodeoxyglucose positron emission tomography or CT (^18^FDG PET-CT) was performed for 35 patients, with positive results for 32 patients (91.4%). Thirty of 105 patients were classified as having possible IE by the modified Duke criteria because of positive echocardiography findings combined with at least 1 minor criterion. Cardiac CT was performed for 9 patients, with positive findings for 8 patients (88.9%). ^18^FDG PET-CT was performed for 2 patients, with positive findings for 1 patient (50.0%). In this possible IE group, IE was yet confirmed as definite by surgical findings combined with positive (prosthetic) valve culture results (27 of 30), polymerase chain reaction testing (2 of 30), or obduction (1 of 30).

Eleven of 105 patients were not suspected to have IE, but they underwent cardiac surgery with intraoperative confirmation of definite IE by the pathologic criteria. Surgery was performed for the following reasons: native valve regurgitation (n = 5), native aortic valve stenosis (n = 2), and prosthesis degeneration (n = 4). Infective endocarditis was suspected for these patients because of valvular perforations and the presence of vegetations. In all 11 patients, at least 2 cultures had positive results (2 valve cultures or a valve culture combined with a culture of a vegetation or a postoperative blood culture). Preoperatively, cardiac CT was performed for 3 patients, but none had positive findings for IE. An overview of the major and minor IE criteria are presented in Table 3.

**Table 3.** Major and Minor Criteria for Patients With Definite and Possible Endocarditis

Criterion	Total cohort, No. (%) (N = 105)
**Total cohort**	
Definite IE according to diagnostic criteria	64 (61.0)
2 Major criteria	54 (51.4)
1 Major criterion and 3 minor criteria	10 (9.5)
5 Minor criteria	0
Major criteria	
Blood culture results positive for IE	54 (51.4)
Echocardiography positive for IE	64 (61.0)
Vegetation	43 (41.0)
>1 cm	14 (13.3)
≤1 cm	29 (27.6)
Abscess	22 (21.0)
Pseudoaneurysm	4 (3.8)
Valvular perforation or new-onset prosthetic valve dysfunction (partial dehiscence or severe regurgitation)	18 (17.1)
Minor criteria	
Predisposition	63 (60.0)
Fever	28 (26.7)
Total vascular phenomena	32 (30.5)
Stroke	19 (18.1)
Myocardial infarction	3 (2.9)
Major arterial emboli	3 (2.9)
Splinter hemorrhage	3 (2.9)
Silent emboli	
Brain	2 (1.9)
Spleen	2 (1.9)
Immunologic phenomena	0
Microbiological evidence not meeting a major criterion	11 (10.5)
Preoperative classified as possible IE and confirmed by culture, histologic examination, or PCR of vegetation, (prosthetic) valve, or abscess	30 (28.6)
**Possible IE**	
1 Major criterion and 1 minor criterion	15 (14.3)
1 Major criterion and 2 minor criteria	15 (14.3)
Major criteria	
Blood culture results positive for IE	30 (28.6)
Echocardiography positive for IE	30 (28.6)
Vegetation	12 (11.4)
>1 cm	6 (5.7)
≤1 cm	6 (5.7)
Abscess	11 (10.5)
Pseudoaneurysm	2 (1.9)
Valvular perforation or new-onset prosthetic valve dysfunction (partial dehiscence or severe regurgitation)	22 (21.0)
Minor criteria	
Predisposition	29 (27.6)
Fever	5 (4.8)
Total vascular phenomena	1 (1.0)
Stroke	1 (1.0)
IE preoperatively not suspected but diagnosed by pathologic criteria and intraoperative confirmation (culture, histologic examination of vegetation, or [prosthetic] valve or abscess)	11 (10.5)

Abbreviations: IE, infective endocarditis; PCR, polymerase chain reaction.

Surgery or reoperation was indicated (class I or IIa^5^) for 88 patients but performed for 80 patients (76.2%). Perioperatively, the most frequently observed findings were (aortic root) abscesses (37 of 80 [46.3%]), prosthetic valve dehiscence (32 of 80 [40.0%]), and vegetations (26 of 80 [32.5%]). Complex anatomical repair of the left ventricular outflow tract, aortic root, or the aortomitral continuity was required for 11 of 80 patients (13.8%). The indications for surgery as well as the types of surgery performed are presented in Table 4. The postoperative 30-day mortality rate was 15.0% (12 of 80), and the 1-year mortality rate was 22.5% (18 of 80). Six of 80 patients (7.5%) who underwent surgery experienced IE recurrence.

**Table 4.** Indications for Surgery and the Types of Surgical Procedures Performed

Indication and type	Patients receiving surgery, No. (%) (n = 80)
Indication	
Heart failure due to (prosthetic) valve dysfunction	22 (27.5)
Uncontrolled infection (abscess, pseudoaneurysm, fistula, or enlarging vegetation)	52 (65.0)
Prevention of embolism	6 (7.5)
Surgery type	
Aortic valve replacement	38 (47.5)
Bentall procedure	20 (25.0)
Mitral valve replacement	7 (8.8)
Mitral valve plasty	2 (2.5)
Aortic replacement plus mitral valve replacement	1 (1.3)
Aortic valve replacement plus mitral valve plasty	3 (3.8)
Aortic valve replacement plus mitral valve replacement plus tricuspid valve plasty	2 (2.5)
Mitral valve replacement plus tricuspid valve plasty	1 (1.3)
Mitral valve replacement plus ascending aorta replacement	1 (1.3)
Bentall procedure plus mitral valve plasty	2 (2.5)
Bentall procedure plus mitral valve replacement	1 (1.3)
Tricuspid valve plasty	1 (1.3)
Isolated ascending aorta replacement	1 (1.3)

Twenty-five of 105 patients were treated conservatively. Despite the indication for surgery, a surgical procedure was not performed for 8 of 25 patients due to comorbidity. Three of these 8 patients died within 30 days after hospital presentation. All 3 had a poor prognosis because of an endocarditis-related stroke. Moreover, 1 of 8 patients in this group experienced PVE recurrence, subsequently requiring surgery because of a stroke. In the other 17 of 25 patients who received conservative treatment, there was no indication for a surgical procedure. No patients in this group died within 1 year after hospital admission, and 5 of 17 patients (29.4%) experienced IE recurrence. These patients had left-sided PVE with small vegetations. Mortality rates as well as *C acnes* recurrence rates are presented in Table 5.

**Table 5.** Mortality and *Cutibacterium acnes* Infective Endocarditis Recurrence Rates for Patients Treated Surgically and Conservatively Across the Indications for Surgery

Outcome	Patients, No. (%)		
	Surgery indicated and performed (n = 80)	Surgery indicated but not performed (n = 8)	Surgery not indicated; only antibiotic treatment (n = 17)
30-d Mortality^a^	12 (15.0)	3 (37.5)	0
1-y Mortality^a^	18 (22.5)	0	0
*C acnes* recurrence	6 (7.5)	1 (12.5)	5 (29.4)
Timeline of recurrence			
During admission	0	0	1 (5.9)
30 d to 3 mo After discharge	2 (2.5)	0	2 (11.8)
3-6 mo After discharge	2 (2.5)	1 (12.5)	0
6 mo to 1 y After discharge	0	0	1 (5.9)
≥1 y After discharge	2 (2.5)	0	1 (5.9)

^a^ For surgically treated patients, postoperative mortality rates are presented. For patients treated conservatively, mortality rates after hospital admission are presented.

All but 1 patient received empirical and, when susceptibility results were available, targeted antibiotic therapy after diagnosis as advised by the European Society of Cardiology guideline.^7^ The latter patient had a severe stroke at presentation with an unfavorable prognosis. The administered intravenous antibiotic regimens included benzylpenicillin, 12 million units/d (71 [67.6%]); benzylpenicillin, 18 million units/d (11 [10.5%]); vancomycin, 2 to 3 g/d (11 [10.5%]); amoxicillin, 12 g/d (6 [5.7%]); and ceftriaxone, 2 to 4 g/d (3 [2.9%]). All 12 patients who experienced *C Acnes* IE recurrence were treated with benzylpenicillin, 12 million units/d. Nineteen patients (18.1%) received additional rifampicin; only 1 of these patients experienced IE recurrence. In accordance with the European Society of Cardiology guideline, intravenous antibiotics were given for at least 6 weeks after the last positive blood or tissue culture result. Two patients (1.9%) did not receive intravenous antibiotic therapy because of patient preference and a chronically infected ascending aorta prosthesis. Clindamycin and amoxicillin, respectively, were given.

## Discussion

To our knowledge, this study is the world’s largest case series of *C acnes* IE. It confirms the hypothesis that the clinical presentation of patients with *C acnes* IE differs significantly from that of other bacterial causative agents. In our study, *C acnes* IE was characterized by absence of fever (66.7%) and close to normal CRP levels. In contrast, in the EURO-ENDO (European Endocarditis) registry, 77% of patients with IE had fever, and the median CRP level was 6.5 mg/dL.^8^ Aside from the diagnostic challenges inherent to PVE,^9^ the diagnostic process in *C acnes* IE is further impaired by the prolonged time to positive blood culture results. So, for male patients with prosthetic heart valves presenting with signs of heart failure or peripheral embolization, there should be a low threshold for performing blood cultures (>5 days’ incubation period). Echocardiography remains the criterion standard in detecting IE, but additional cardiac CT or ^18^FDG PET-CT is useful in suspected PVE with periannular extension.^9,10^ For cardiac CT and ^18^FDG PET-CT, we observed a sensitivity of 85.0% and 89.2%, respectively. Thus, the high glucose uptake in PET-CT does not match the assumed low virulence of *C acnes* IE.

We hypothesize that *C acnes* IE occurs mostly among male patients because the sebaceous glands, where *C acnes* predominates,^11^ proliferate under stimulation of androgens.^12^ Because the male chest is rich with sebaceous glands, we suggest the surgical wound is contaminated with *C acnes* during index (valve) surgery. Subsequently, we assume the low virulence and slow growth of *C acnes*^13^ results in patients presenting several years after index surgery. We hypothesize that additional preoperative measures, such as topical agents, can be effective in *C acnes* IE prevention for patients with acne on the thorax. However, further research is needed on this topic.

Although levels of inflammatory markers are generally low at presentation, the high share of aortic root abscesses emphasizes the indolent but not innocent nature of *C acnes* IE. If surgery is indicated,^7^ it should be offered to patients because mortality is high when it is indicated but not performed.^14,15^ In accordance with the current guideline, conservative treatment should be performed in right-sided IE or native valve IE without uncontrolled infection, heart failure, or large vegetations. In PVE with small vegetations, however, antibiotic treatment alone seems insufficient to eradicate *C acnes*, considering the relatively high rates of short-term IE recurrence. More research is needed on the optimal antibiotic therapy, with or without adding rifampicin. Moreover, further research should explore the effectiveness of chronic oral suppression therapy in *C acnes* IE. For PVE with small vegetations, we suggest a new class IIa indication for surgery for this group.

### Limitations

Our study has several limitations. First, the retrospective design of our study creates the risk of information bias. Although we comprehensively assessed all patients’ records to and from referring hospitals, we cannot rule out possible inaccuracies in the provided data. Second, because we opted not to perform statistical analysis because of the small sample size, we cannot provide the best antibiotic regimen (with or without adding rifampicin) to treat *C acnes* IE and to minimize the risk of IE recurrence. Also, although we observed a trend of higher *C acnes* IE recurrence rates after conservative treatment, we must look at this finding in the light of a lack of underlying statistical evidence. Third, *C acnes* is a known skin contaminant, so blood or tissue culture results could have been false positive. Nevertheless, all included cases had multiple positive blood or tissue culture results, combined with positive echocardiography findings and/or surgical evidence, which means that all cases were definite IE by *C acnes*.

## Conclusions

In this case series, we found that *C acnes* endocarditis was seen predominantly among male patients with prosthetic heart valves. Diagnosing *C acnes* endocarditis is difficult due to its atypical presentation, with frequent absence of fever and inflammatory markers. The prolonged time to positivity of blood culture results further delays the diagnostic process. Not performing surgery when indicated seems to be associated with higher mortality rates. However, each patient’s operability must always be taken into account. For patients with PVE with small vegetations, there should be a low threshold for surgery because these patients seem to be prone to short-term endocarditis relapse.

## Notes

Supplement.
Data Sharing Statement
