# Supplementary material for: Clinical Characteristics and Outcomes of Patients With Cutibacterium acnes Endocarditis
Source: JAMA Netw Open. 2023 Jul 12;6(7):e2323112. doi: 10.1001/jamanetworkopen.2023.23112 (PMC10339155; doi:10.1001/jamanetworkopen.2023.23112)
Supplement: Supplement. — Data Sharing Statement [file jamanetwopen-e2323112-s001.pdf]

# Data Sharing Statement

Heinen. Clinical Characteristics and Outcomes of Patients With Cutibacterium acnes Endocarditis. *JAMA Netw Open*. Published July 12, 2023.
doi:10.1001/jamanetworkopen.2023.23112

**Data**
**Data available:** No
